# Towards an understanding of the multilevel factors associated with maternal health care utilization in Uttar Pradesh, India

**DOI:** 10.1080/16549716.2017.1287493

**Published:** 2017-07-06

**Authors:** Sanjeev Sridharan, Arnab Dey, Aparna Seth, Dharmendra Chandurkar, Kultar Singh, Katherine Hay, Rachael Gibson

**Affiliations:** ^a^ The Evaluation Centre for Complex Health Interventions, St. Michael’s Hospital, University of Toronto, Toronto, ON, Canada; ^b^ Sambodhi Research & Communications Pvt. Ltd, Noida, UP, India; ^c^ Bill and Melinda Gates Foundation, Delhi, India

**Keywords:** Maternal health, health inequities, evaluation

## Abstract

**Background:** This paper explores the multilevel factors associated with maternal health utilization in India’s most populous state, Uttar Pradesh. 3 key utilization practices: registration of pregnancy, receipt of antenatal care, and delivery at home are examined for district and individual level predictors. The data is based on 5666 household surveys conducted as part of a baseline evaluation of the Uttar Pradesh Technical Support Unit (UPTSU.) program.

**Objectives:** This intervention aims to assist the Government of Uttar Pradesh in increasing the efficiency, effectiveness, and equity of service delivery across a continuum of reproductive, maternal, new-born, child, and adolescent health (RMNCH+A) outcomes.

**Methods:** The paper employs multilevel models that control for individuals being nested within districts in order to understand the predictors of maternal health care utilization.

**Results:** The study identifies several individual-level predictors of health care utilization, including: literacy of the woman, the husband’s schooling, age at marriage, and socio-economic factors. Key predictors of pregnancy registration include husband’s schooling (OR 1.49, 95% CI 1.26–1.76), having a bank account (OR 1.36, 95% CI 1.11–1.68), and owning a house (OR 2.28, 95% CI 1.85–2.80). Factors affecting antenatal care include the woman’s literacy (OR 1.49, 95% CI 1.28–1.73), the respondent having had a job in the last year (OR 1.39, 95% CI 1.10–1.77), and owning a house (OR 2.83, 95% CI 2.27–3.53). Home delivery tends to be associated with woman’s literacy (OR 0.62, 95% CI 0.54–0.72) and marriage age of 15 and younger (OR 1.48, 95% CI 1.26–1.73).

**Conclusions:** Interventions having equity considerations need to disrupt existing patterns of the health gradient. Successful implementation of such interventions, necessitate understanding the mechanisms that can disrupt the unequal utilization patterns and target domains of disadvantage. Knowledge of key predictors of utilization can aid in the implementation of such complex interventions.

## Background

This paper explores the factors that impede or facilitate the utilization of health care services. Three specific utilization practices are explored in India’s most populated state, Uttar Pradesh (UP): registration of the pregnancy, receiving any antenatal care, and whether the delivery was at home. Predictors of utilization include literacy of the mother, husband’s schooling, age at first marriage, and socio-economic factors. Knowledge of such predictors can help implement complex interventions that can incorporate equity considerations in health care utilization.

UP is India’s most populous state. As in other settings in India, maternal health utilization in UP is driven by a complex set of factors that include caste, religion, poverty, literacy, geography, and cultural norms. This paper explores the impacts of some of these factors on maternal health care utilization in UP (Please note: by utilization we mean the self-reported utilization of the health system.)

In order to improve the health outcomes of the state and reduce health inequities in service delivery and utilization, the Government of Uttar Pradesh (GoUP) and the Bill and Melinda Gates Foundation (BMGF) came together to form the Uttar Pradesh Technical Support Unit (UPTSU). The purpose of this unit is to support the State Government’s efforts to increase the efficiency, effectiveness, and equity of service delivery across a continuum of reproductive, maternal, child, and adolescent health (RMNCH+A) outcomes in UP [1].

Any intervention seeking to impact maternal health outcomes must come to terms with the complexities [2–4] of the health care utilization process. Interventions do not work in a vacuum. Therefore, understanding the complexities and the precise mechanisms by which the drivers of health care utilization can be modified becomes critical to the success of an intervention.

Maternal health care utilization is not simply a matter of access, choice, or needs; rather, it can be shaped by individual and cultural-level expectations, constraints, and contexts [5–10]. The utilization of maternal health care can further be driven by multilevel factors, such as those operating at the individual, community, and district levels [5,11–13]. Cultural norms within the community or district, for example, can hinder a woman from seeking or receiving health care. These cultural norms can also take place at the level of the individual, where a woman’s personal beliefs or her family’s belief system can either facilitate or impede the process of seeking care [6,8,10].

This paper addresses the following central question: ‘Why do some individuals utilize or not utilize the formal health system?’ In order to provide a compelling response to this question, it is important to develop an understanding of the social determinants of health [14,15]. We must look beyond the realm of individual preferences to explore the various constraints and contextual factors operating at multiple levels.

Our primary objective is to understand the factors that enable or impede the utilization of health care services. To this end, three specific utilization practices are explored: the registration of a woman’s pregnancy, whether the women received any antenatal care, and whether the delivery of the child was at home. This paper uses multivariate statistical techniques [16] to understand the factors associated with maternal health utilization. While the paper seeks to understand the ‘problem space’ [17] of inequities in health care utilization, the evaluation also provides us with an opportunity to develop knowledge about the ‘solution space’ [17] of such inequities. In this respect, we are keen to develop insights into what has worked for whom [18] to address gradients in the utilization of the health system.

#### Context of study

With a population of over 210 million [19], UP is the most populous state in India with a recorded growth rate of 20.2% in the last decade. The majority of the state’s population consists of Hindus, at 79.7%, while Muslims comprise 19.3% of the population [19]. A strong social hierarchy also exists in the state, with 25% of the population being Scheduled Caste (SC) [20] and 50% of the population belonging to Other Backward Classes (OBC) [20], that is, backward castes identified by the Government of India that are not included in the list of Scheduled Castes [21,22].

While constituting about 16.5% of the national population, UP also adds significantly to the health burden of the nation. The state reports disproportionately higher rates of maternal mortality: 392 deaths compared to the national figure of 178 deaths per 100,000 live births – as well as infant mortality at 50 deaths per 1000 live births compared to the national rate of 40 [23]. In addition to the poor status of these health indicators, the status of public health care is also characterized by disparities in the access and utilization of health care services. National-level studies indicate that religion, social hierarchy, literacy, and economic status can influence an individual’s access to and utilization of health care services [20]. Although it is important for UP to improve the status of its health outcomes in order to assist the nation in realizing its health outcomes goals, the emphasis on health outcomes must also be accompanied by a focus on reducing existing health inequities in the state with a view to delivering on its commitment to ‘health for all’ [24,25].

#### The UPTSU intervention

The Technical Support Unit (TSU) provides techno-managerial support to improve the efficiency, effectiveness, and equity of delivery of key RMNCH+A interventions. It does so by establishing improved internal accountability coupled with enhanced techno-managerial skills and capabilities. The TSU supports the GoUP in implementing the National Rural Health Mission (NRHM) RMNCH+A strategy [26] and in unlocking its ‘execution capacity’. The TSU works in 100 blocks of the 25 most underprivileged districts of the state jointly identified by the Government of India, the GoUP, and the BMGF

#### Addressing the social determinants of health

**Figure 1.**
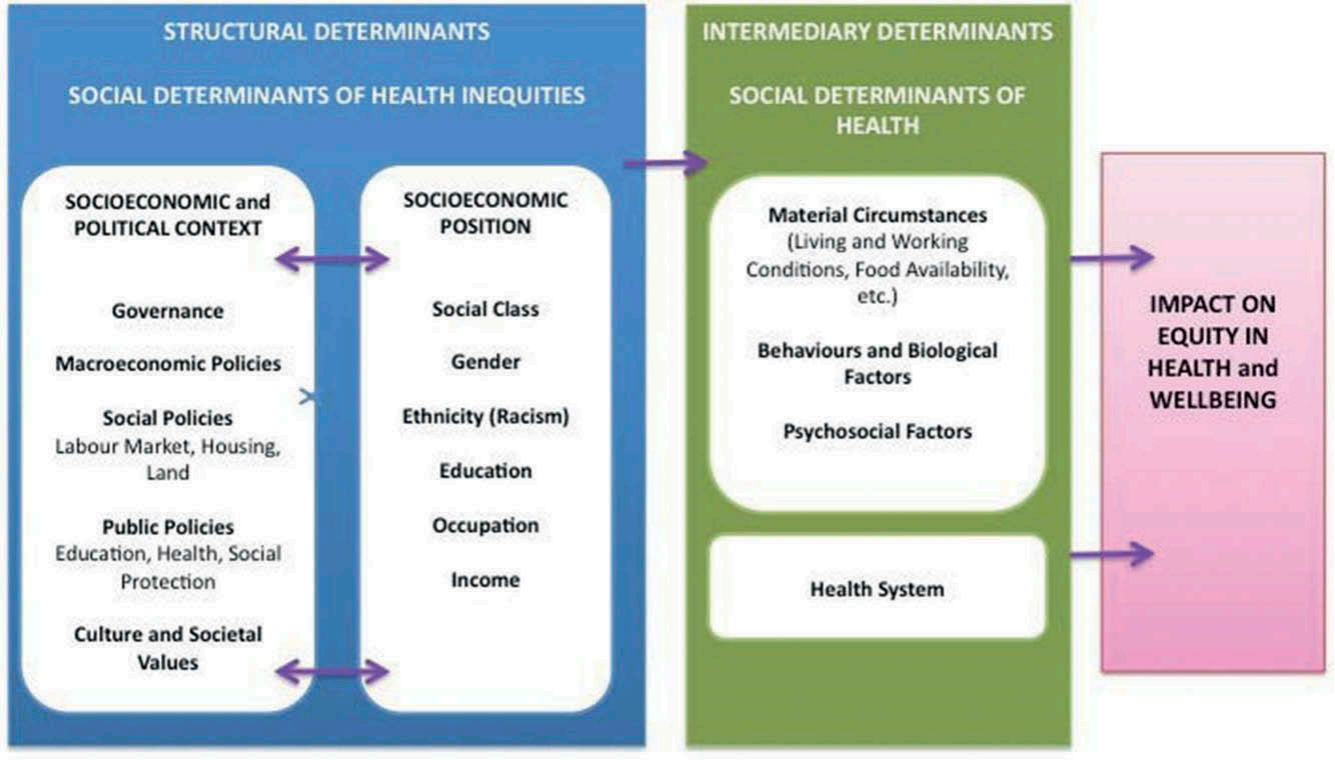
Social Determinants of Health framework.

The Social Determinants of Health framework (SDH) developed by the World Health Organization (WHO) discusses multiple drivers that might impact health care utilization [27,28]. Solar and Irwin [27], for example, suggest that a social determinants of health strategy needs to address the multiple ‘structural’ and ‘intermediary’ factors associated with health and health inequities (see Figure 1). As described in Figure 1, key structural drivers include the socio-economic and political context, socio-economic position, social class, gender, ethnicity, education, occupation, and income. Intermediary drivers include material circumstances, behavioural and biological factors, psychosocial factors, and health system drivers. Many of these drivers can operate at multiple levels. As an example, a low individual-level socio-economic position can serve as a disadvantage when it comes to seeking and receiving health care. This can be further exacerbated if the individual also resides in a poor community. The cumulative effect of the multiple levels of disadvantage can further reduce access to health care [29].

As Solar and Irwin [27] point out, it is important to consider both structural and intermediary factors because this promotes a focus on the ‘root causes’ of health and health equity outcomes: ‘The vocabulary of “structural determinants” and “intermediary determinants” underscores the causal priority of the structural factors’ ([27], p. 67).

Solar and Irwin [27, p. 68] also encourage us to focus on how an intervention plans to address the structural determinants of health outcomes:

In conventional usage, the term ‘social determinants of health’ has often encompassed only intermediary determinants. However, interventions addressing intermediary determinants can improve average health indicators while leaving health inequities unchanged. For this reason, policy action on structural determinants is necessary. To achieve solid results, SDH policies must be designed with attention to contextual specificities, which should be rigorously characterized using methodologies developed by social and political science.

It is worth reiterating that policies focusing on health inequities might need to consider how an intervention can improve average health outcomes while at the same time exacerbating health equity outcomes. As Graham [30, p. 101] argues: ‘the social factors promoting and undermining the health of individuals and populations should not be confused with the social processes underlying their unequal distribution. This distinction is important because, despite better health and improvement in health determinants, social disparities persist’.

## Methods

### Sampling

The baseline study involved a community-based household survey in 200 blocks in the state (100 project blocks and 100 additional blocks matched *a priori* for comparison). The 200 project blocks were spread across 49 districts. The Primary Sampling Unit (PSU) for the study was the Accredited Social Health Activist (ASHA) catchment area, with a population of about 1000. All districts of the state were ranked on a composite index of indicators comprising: the maternal mortality ratio (MMR), percentage of safe deliveries, infant mortality rate (IMR), percentage of children 12–23 months fully immunized, total fertility rate (TFR), and contraceptive prevalence rate (CPR) – modern method, with the 25 lowest-performing districts designated as High Priority Districts (HPD). A multistage sampling design was used to obtain a representative sample of live births in the last 12 months from the 25 HPD of UP

The survey aimed to reach the beneficiaries of the RMNCH+A delivery system and included a number of target groups for the purpose described later in this paper:

- Women with a child aged 0–5 months
- Women with a child aged 6–11 months
- Women with a child aged 12–23 months
- Currently married women in the age group of 15–49 years
- Adolescent girls in the age group of 10–19 years
- Women that had a miscarriage or still birth one year prior to the survey
- Women that had a neo-natal death in the year prior to the survey.

A sampling of households began with a complete listing of all the households in a PSU to identify the eligible target group for the study. Simple random sampling was then used to select the required number of individuals within a PSU from the list of eligible respondents identified from the listing exercise. A total of 15,164 women were interviewed from all 7 categories. The present paper only focuses on women with a child under 1 year of age at the time of the interview. A total of 5666 such women were enrolled in the study from 600 PSU spanning 200 blocks and 49 districts.

Our study’s protocols were reviewed and approved by the NRHM of UP, PHS-ERB (an independent ethical review board), and the Health Ministry Screening Committee’s (HMSC) Indian Council for Medical Research (ICMR). These protocols were also registered with the Clinical Trial Registry – India (CTRI/2015/09/006219).

### Variables

#### Dependent variables

The three dependent variables in this study are: registration of pregnancy, receipt of antenatal care, and home delivery. Registration of pregnancy ensures that a pregnant woman is included in the public health system to receive services. Receipt of antenatal care is also included as a dependent variable, as it indicates the initiation of access to public health care services by an expecting mother. Women delivering at home are also treated as a dependent measure because home delivery can imply being ‘left-out’ of the formal services and care provided by health facilities.

#### Independent variables

The variables included in the model are informed by the social determinants of health [27]. The independent variables include measures of both the intermediate and structural determinants of health. At the individual level, our independent variables include measures of: religion, caste, economic status, whether a woman is empowered to make decisions by herself, literacy, age at the time of the interview, and age at first marriage.

The study includes key measures related to the economic status of the women. The first of these measures consists of a wealth index, which is a continuous measure of wealth based on a principal component analysis. This index includes measures of household-level assets as well as household characteristics. A second measure related to economic status probes whether the respondent held a job during the last year (7% of the sample had been paid for a job outside of their own household work). A third measure focuses on whether the respondent or a member of the household has a bank or post office account (89% responded ‘yes’). And a fourth measure looks at whether the respondent or respondent’s household owns a house (85% of respondents or respondents’ households owned their house).

The study also includes measures related to religion and caste. Given that Hindus represent the majority of the sample (82%), a binary variable measuring the respondent’s religion is included in the model. Caste is measured using two binary measures. The first is whether the respondent belongs to a Scheduled Caste or Scheduled Tribe (SC/ST) (30% of the sample belongs to a SC/ST). The second is whether the respondent is of ‘Other Backward Class’ (OBC) (50% of the sample is of ‘OBC’).

We include two measures of a woman’s autonomy to make her own decisions. The first is whether the respondent makes her own decisions about health care (60% of the sample indicated that they made their own decisions about health care). The second is whether the respondent is allowed to go to the health clinic by herself (only 19% of the sample indicated that they are allowed to go to the health clinic by themselves).

The study measures women’s literacy using a variable that explores whether the respondent can read or write (44% of the respondents said they could read or write). We also include a measure of the husband’s schooling, which explores whether he has ever attended school (75% of husbands indicated that they had attended some school).

The study includes a measure of age at first marriage. For a majority (61%) of the sample, age at first marriage was 15 or younger. Child marriages are prevalent in the state of UP We, therefore, created a binary variable that measures whether the age at the time of first marriage was 15 years or younger.

The model includes a number of district-level variables to capture the context of health care utilization. These include the average distance of the villages from the district headquarters (which serves as a measure of access to the district hospital), as well as separate contextual measures of the density of community health centres, primary health centres, and sub-centres. Other district-level measures include the proportion of the population that are SC/ST and the proportion of the population that are illiterate. From the social determinants of health perspective, being illiterate or of a lower caste can serve as a barrier to health care. We explore the association between district-level measures of illiteracy and caste and health utilization at the individual level. In addition, the model includes a district-level measure of the proportion of the population that is female. This measure is included for exploratory purposes, as it could potentially measure multiple dimensions, such as the migration of men from the district and existing cultural norms within the districts that favour preferences for sons (Table 1).

**Table 1.** Descriptive statistics for dependent and independent variables.

	Level	Mean	SD	N
Dependent variables				
Pregnancy registration	Individual	0.71	0.46	5666
Antenatal care	Individual	0.51	0.50	5666
Delivery at home	Individual	0.32	0.47	5666
Independent variables				
Infrastructure				
Average distance of villages to district headquarters	District	34.36	4.97	49
Community health centres (numbers/10,000 population)	District	0.03	0.02	49
Primary health centres (numbers/10,000 population)	District	0.11	0.03	49
Sub-centres (numbers/10,000 population)	District	0.08	0.08	49
Demographics				
Age at interview	Individual	26.64	4.65	5666
Married at 15 years or less	Individual	0.61	0.49	5666
Hindu	Individual	0.82	0.38	5666
SC/ST	Individual	0.30	0.46	5666
OBC	Individual	0.57	0.49	5666
Proportion females	District	47.64	0.93	49
Proportion SC/ST	District	21.40	6.18	49
Literacy				
Can read or write	Individual	0.44	0.50	5666
Husband ever attended school	Individual	0.75	0.43	5666
Proportion of illiterates in a district	District	45.1	6.70	49
Prosperity				
Own a house	Individual	0.85	0.36	5666
Own a bank account	Individual	0.89	0.32	5666
Members per room in a household	Individual	0.39	0.30	5666
Worked in the last 1 year	Individual	0.07	0.26	5666
Wealth index	Individual	0.01	3.02	5666
Access to health care & decision making				
Health decisions taken by self	Individual	0.6	0.49	5666
Health care can be accessed by self	Individual	0.19	0.39	5666

### Statistical analysis

Given the clustering of individuals within districts, it was necessary to develop multilevel statistical models for our analysis [8,12,16]. Multilevel modeling controls for potential clustering effects and corrects for any bias in the standard errors [16]. As each of the dependent variables is binary in nature, we ran separate multilevel logistic regression models for each of the three dependent variables.

A four-step model was implemented in developing the multilevel model [12]. First we ran a model for each of the dependent variables without including any covariates. This first model represents the total variance in maternal health utilization for each of the dependent variables between the districts. Models 2 and 3 included only individual- and district-level factors, respectively. In model 4, both individual- and district-level factors were added. Due to spatial considerations, the present study only reports models 1 and 4.

## Results

### Variations in health care utilization practices across district*s*

**Figure 2.**
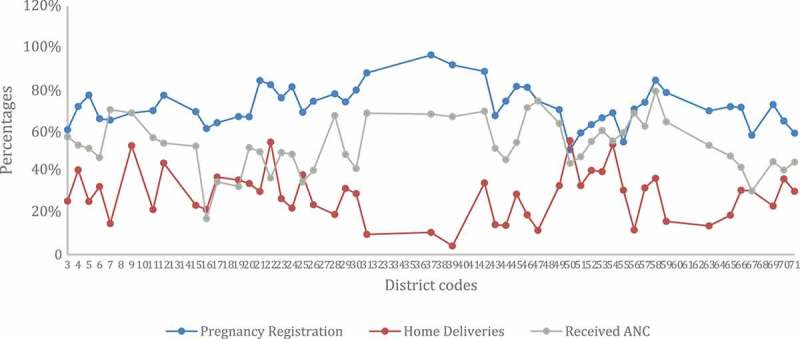
Variations in pregnancy registration, receipt of antenatal care (A.N.C.), and delivery at home across districts.

We first explore variations in each of the utilization practices across the districts. Figure 2 describes the variation in each of the three utilization measures. Variations are evident in the utilization of maternal health care across the districts.

As a first step, we assess whether the data support the decision to model the multiple levels of district and individuals. The variance components for each of the null models (models without any covariates) are described in Table 2. In each case, the variance components corresponding to the district level are significant, indicating that there is variation across the districts for each of the maternal health utilization variables.

**Table 2.** Variance components corresponding to the null model.

	Register	Home	Antenatal care
District intercept coefficient	0.92***	–0.88***	0.09
(standard error)	(0.06)	(0.08)	(0.07)
Variance component	0.14***	0.29***	0.18***
Chi-square	206.7	420.5	263.6

***p < 0.001.

#### Multivariate analysis

Tables 3–5 describe the results of the multivariate multilevel models corresponding to the three utilization practices. Please note that the test statistic is the *t*-ratio formed by dividing the estimated regression coefficient by its standard error. Key predictors of registration include measures of education, economic status, age at first marriage, and living arrangements. The husband of the respondent having any schooling enhances the odds of the woman being registered by 49%. The respondent’s ability to read or write also increases the odds of being registered – this time by 28%. The odds of registering jump by a remarkable 128% if the respondent owns a house, whereas having a bank account increases the odds of registering by 36%. We see a decrease in the odds of registering by 16% if the respondent’s age at first marriage was 15 years or less. As described in greater detail in what follows, this finding indicates that marriage age may be a marker for women’s autonomy to make health care decisions. Increased density in living arrangements is also associated with decreased odds of registering. Increased density in living arrangements might also be indicative of greater poverty.

**Table 3.** Multilevel models for pregnancy registration.

	B (Std. error)	Odds ratio	95% confidence interval	*t*-test
***District-level factors***				
Distance	0.00	1.00	(0.99, 1.02)	0.43
	(0.01)			
Proportion female	–0.03	0.98	(0.88, 1.08)	–0.49
	(0.05)			
Proportion SC/ST	0.01	1.01	(0.99, 1.03)	1.50
	(0.01)			
Proportion illiterate	0.00	1.00	(0.98, 1.01)	–0.35
	(0.01)			
Community health centres	–4.39	0.01	(0.00, 6.74)	–1.41
	(3.12)			
Primary health centres	1.58	4.87	(0.13, 176.6)	0.89
	(1.78)			
Sub-centres	2.49	12.01	(2.13, 67.86)	2.90***
	(0.86)			
***Individual-level factors***				
Hindu	–0.02	0.98	(0.79, 1.22)	–0.17
	(0.11)			
SC/ST	0.19	1.21	(0.99, 1.47)	1.92
	(0.10)			
OBC	0.00	1.00	(0.82, 7.23)	0.03
	(0.10)			
Own house	0.82	2.28	(1.85, 2.80)	7.79***
	(0.11)			
Bank account	0.31	1.36	(1.11, 1.68)	2.93***
	(0.11)			
Self health	0.17	1.18	(0.94, 1.49)	1.42
	(0.12)			
Health alone	–0.19	0.82	(0.67, 1.01)	–1.86
	(0.10)			
Read or write	0.24	1.28	(1.12, 1.46)	3.53***
	(0.07)			
Any job in the last year?	0.09	1.09	(0.83, 1.45)	0.62
	(0.14)			
Wedding below the age of 15	–0.18	0.84	(0.74, 0.96)	–2.63***
	(0.07)			
Husband schooling	0.40	1.49	(1.26, 1.76)	4.69***
	(0.08)			
Density	–0.26	0.77	(0.62, 0.96)	–2.37**
	(0.11)			
Wealth index	0.03	1.03	(0.99, 1.06)	1.81
	(0.02)			
Age at interview	–0.01	0.99	(0.98, 1.01)	–0.75
	(0.01)			

***p < 0.01; **p < 0.05.

**Table 4.** Multilevel model for antenatal care.

	B (Std. error)	Odds ratio	95% confidence interval	*t*-test
***District-level factors***				
Distance	–0.01	0.99	(0.97, 21.01)	–0.61
	(0.01)			
Proportion female	0.32	1.38	(1.23, 21.55)	5.73***
	(0.06)			
Proportion SC/ST	0.00	1.00	(0.99, 21.02)	0.51
	(0.01)			
Proportion illiterate	0.01	1.01	(0.99, 21.03)	0.92
	(0.01)			
Community health centres	–4.84	0.01	(0.00, 7.33)	–1.43
	(3.39)			
Primary health centres	–2.61	0.07	(0.00, 3.20)	–1.40
	(1.87)			
Sub-centres	2.30	9.98	(2.29, 43.54)	3.15***
	(0.73)			
***Individual-level factors***				
Hindu	–0.14	0.87	(0.72, 1.06)	–1.38
	(0.10)			
SC/ST	–0.24	0.79	(0.61, 1.01)	–1.86
	(0.13)			
OBC	–0.32	0.73	(0.58, 0.92)	–2.68***
	(0.12)			
Own house	1.04	2.83	(2.27, 3.53)	9.15***
	(0.11)			
Bank account	0.10	1.11	(0.90, 1.37)	0.95
	(0.11)			
Self health	–0.07	0.93	(0.82, 1.05)	–1.18
	(0.06)			
Health alone	–0.06	0.94	(0.81, 1.10)	–0.78
	(0.08)			
Read or write	0.40	1.49	(1.28, 1.73)	5.17***
	(0.08)			
Any job in the last year?	0.33	1.39	(1.10, 1.77)	2.73***
	(0.12)			
Wedding below the age of 15	–0.34	0.71	(0.63, 0.81)	–5.22***
	(0.07)			
Husband schooling	0.27	1.30	(1.15, 1.48)	4.08***
	(0.07)			
Density	–0.17	0.85	(0.66, 1.08)	–1.36
	(–0.12)			
Wealth index	0.10	1.11	(1.08, 1.13)	8.25***
	(0.01)			
Age at interview	–0.03	0.97	(0.96, 0.98)	–5.21***
	(–0.01)			

***p < 0.01; **p < 0.05.

**Table 5.** Multilevel model for home delivery.

	B (Std. error)	Odds ratio	95% confidence interval	*t*-test
***District-level factors***				
Distance	0.05	1.05	(1.03, 1.08)	3.99***
	(0.01)			
Proportion female	–0.11	0.90	(0.74, 1.08)	–1.15
	(0.09)			
Proportion SC/ST	–0.04	0.96	(0.94, 0.98)	–4.31***
	(0.01)			
Proportion illiterate	0.00	1.00	(0.99, 1.02)	0.33
	(0.01)			
Community health centres	–2.00	0.14	(0.00, 34.19)	–0.73
	(2.74)			
Primary health centres	–0.22	0.80	(0.00, 137.5)	–0.09
	(2.55)			
Sub-centres	–2.62	0.07	(0.01, 0.42)	–3.04***
	(0.86)			
***Individual-level factors***				
Hindu	–0.03	0.97	(0.82, 1.15)	–0.35
	(0.09)			
SC/ST	0.54	1.72	(1.35, 2.19)	4.43***
	(0.12)			
OBC	0.53	1.69	(1.30, 2.21)	3.93***
	(0.13)			
Own house	–0.09	0.92	(0.78, 1.08)	–1.04
	(0.08)			
Bank account	–0.24	0.79	(0.65, 0.95)	–2.50**
	(0.10)			
Self health	–0.02	0.98	(0.88, 1.09)	–0.42
	(0.06)			
Health alone	–0.01	0.99	(0.84, 1.16)	–0.11
	(0.08)			
Read or write	–0.47	0.62	(0.54, 0.72)	–6.36***
	(0.07)			
Any job in the last year?	0.29	1.33	(1.02, 1.74)	2.12**
	(0.14)			
Wedding below the age of 15	0.39	1.48	(1.26, 1.73)	4.87***
	(0.08)			
Husband schooling	–0.13	0.88	(0.76, 1.01)	–1.84
	(0.07)			
Density	–0.20	0.82	(0.65, 1.03)	–1.72
	(0.12)			
Wealth index	–0.04	0.96	(0.94, 0.99)	–2.99***
	(0.01)			
Age at interview	0.01	1.01	(0.99, 1.02)	1.26
	(0.01)			

***p < 0.01; **p < 0.05.

For the most part, the district-level factors are not significantly related to registration. The one exception, here, is the measure of sub-centres. We find a statistically significant increase in the odds of registration due to the presence of sub-centres.

#### Antenatal care

Key predictors of receiving antenatal care include caste, education, economic status, age at marriage, and age at interview. Being of ‘OBC’ is associated with reduced odds of receiving antenatal care by 27%. If the respondent’s husband has any schooling, the likelihood of the respondent receiving antenatal care increases by 30%. The respondent’s ability to read or write is associated with increased odds of receiving antenatal care by 49%. If the respondent has held a job within the past one year, the odds of receiving antenatal care increase by 39%. Owning a house increases the odds of the respondent receiving antenatal care by a remarkable 183%. An increase in the wealth index is also associated with a greater likelihood of receiving antenatal care. If the respondent’s age at first marriage was 15 years or younger, the odds of receiving antenatal care are reduced by 29%. An increased age at the time of the interview is also associated with a reduced likelihood of receiving antenatal care.

District-level factors that are predictive of receiving antenatal care include the proportion of the population that are female and the density of sub-centres. An increase in the proportion of the district population who are females is associated with increased odds of receiving antenatal care. An increase in the density of sub-centres is also associated with increased odds of receiving antenatal care.

#### Home delivery

Caste, education, economic status, and age at first marriage are all associated with home delivery. The respondent being SC/ST or ‘OBC’ substantially increases the odds of home delivery (by 72 and 69%, respectively). If the respondent is able to read or write, the likelihood of having a home delivery is reduced by 38%. If the respondent’s husband has any schooling, the odds of having a home delivery are also reduced, by 12%. Having a bank account reduces the likelihood of having a home delivery by 21%. An increase in the wealth index is also associated with a decrease in the odds of home delivery, whereas the likelihood of home delivery increases by 48% if the respondent’s age at first marriage was 15 years or lower.

The study finds three district-level contextual factors to be statistically significant predictors of home delivery. Increased distance to the headquarters is associated with a greater likelihood of home delivery, whereas an increase in the proportion of SC/ST and an increase in the number of sub-centres are both associated with reduced odds of home delivery. The implications of the contextual factors will be discussed at greater length in the next section of the paper.

## Discussion

The three utilization variables explored in this paper are crucial to improving maternal and newborn health. These three variables also have a causal relationship among them – early registration of pregnancy ensures that the health system has sufficient time to provide antenatal care to the pregnant woman and also maximizes the window of opportunity for health care providers to counsel and motivate women to opt for an institutional delivery. An increased number of antenatal check-ups of pregnant women also improves the likelihood of identifying pregnancy-related complications, such as high or low blood pressure, low haemoglobin count, or high protein content in urine. Early identification of pregnancy-related complications creates opportunities for improved management of such problems, thereby leading to better maternal and newborn health outcomes.

With respect to all three utilization variables, our findings highlight the influential role of a woman’s husband. The study suggests that when a woman’s husband has received any schooling, she is much more likely to register her pregnancy, receive antenatal care, and choose an institutional delivery. Moreover, women who are able to read or write have better chances of receiving antenatal care and higher numbers of them choose to deliver at health facilities rather than at home. These two findings are consistent with the results in the literature finding that education matters for maternal health utilization [9,31–35].

One intriguing result of our research pertains to the predictive power of the variable measuring age at first marriage – specifically, whether the woman was 15 years or younger. This variable is associated with decreased odds of registration and antenatal care, as well as increased odds of home delivery. Given that child marriages are prevalent in UP, these patterns of results might suggest the impacts of child marriage. Mechanisms by which age at first marriage might impact maternal health utilization can perhaps be explained by such observations as ‘women who marry early often leave school, do not work outside the home and have restricted mobility’ [36]. The complex and reinforcing nature of such factors represents a formidable challenge for interventions seeking to address inequities and impact health care utilization outcomes.

Caste also matters as a predictor of health utilization. As described in the SDH framework, caste is an important structural determinant. Being from an ‘OB.C.’ or from a SC/ST increases the odds of a home birth and significantly reduces the likelihood of receiving antenatal care [9,37–40].

The findings also indicate the important effects of economic status on health utilization. Women belonging to families who own a house are more likely to register their pregnancies and receive antenatal services. We also find that women who belong to wealthier households have increased odds of receiving antenatal care and delivering at health facilities rather than at home. In addition, women belonging to households having a bank account are more likely to register their pregnancy and have an institutional delivery.

### District-level contextual effects

One important caveat needs to be noted in interpreting the district-level contextual effects: statisticians have cautioned that the contextual-level effects in multilevel models should not be treated as ‘causal effects’ and recommend viewing such results as descriptive or exploratory [41]. With this caveat noted, the study finds interesting patterns of results for the following three district-level measures: density of sub-centres, distance to central headquarters, and proportion of the population that is female. The density of sub-centres is strongly predictive for all three health care utilization outcomes. An increase in the density of sub-centres is associated with greater odds of registration and antenatal care and reduced likelihood of delivery at home. The distance to central headquarters represents a significant predictor of delivery at home, but not a significant predictor of receipt of antenatal care or pregnancy registration. An increase in the proportion of the population that is female is associated with an increase in the odds of women utilizing antenatal care. At present, given the aforementioned methodological caveat, these results should be viewed in a more exploratory vein. Further detailed study is needed to demonstrate their potential causal implications.

#### Implications of the results for implementing interventions

Key implications of the observed patterns of results include the following:

1. The analysis presented in this paper identifies a number of factors that are predictive of health utilization, including women’s literacy, the husband’s schooling, caste, and age at first marriage. A health utilization gradient can be associated with each of these factors. Changing patterns of equities implies that interventions need to disrupt the existing patterns of the health gradient in order to be effective.
2. The multilevel models developed in this paper explore the direct effects for the various factors. An important area of research on inequities coming out of the feminist research literature engages the concept of intersectionality [42–44]. Intersectionality is defined by Bowleg [44] as ‘a theoretical framework for understanding how multiple social identities such as race, gender, sexual orientation, SES [socioeconomic status], and disability intersect at the micro level of individual experiences to reflect interlocking systems of privilege and oppression’ (p. 1267). Based on this concept, an example of a guiding question for implementing interventions might be: What specific processes will interventions develop to reach the women and families who are located at the intersection of multiple categories of disadvantage? In other words, what will the program do to reach women who, for example, cannot read or write, whose husbands have no schooling, are from lower castes, and were married at 15 or earlier?

#### Limitations

It is important to mention the following limitations of the paper:

- One key limitation from a multilevel perspective is that the district might be too ‘coarse’ a level at which to observe contextual effects. Other finer levels, such as blocks or the community, might be preferable for future investigation. However, the advantage of the district level is the availability of district-level data to explore the context. We are presently in the process of developing multilevel models at the block level.
- Whereas the developed multilevel models explore the direct effects of the different social determinants of health, the interactions between the different social determinants of health are not explored in this study. Future multilevel models that we plan to develop as part of our research project will explore the interactions between the various factors.

## Conclusion

This paper explores the factors associated with three health care utilization practices. The obtained patterns of results are consistent with the predictions of the SDH framework [27]. Examples of individual-level variables that strongly predict utilization practices include caste, economic status, the woman’s literacy, the husband’s schooling, and the respondent’s age at first marriage. While the findings are not surprising, the pattern of observed results sheds light on the challenges any intervention will face when it comes to disrupting existing utilization patterns in order to address inequities in health utilization.

This paper identifies a number of factors that might serve to both facilitate and hinder maternal health utilization. These predictors of utilization practices help to isolate factors that contribute to inequities in health utilization. The UPTSU program has the potential to address these inequities in utilization through its key activities at the community, facility, and systems levels. The evaluation needs to empirically test the role of UPTSU interventions in addressing existing inequities in utilization as well as the causal impacts of the program on health care utilization patterns. The notion of intersectionality may be useful, here, in preparing future interventions for the challenge that lies ahead with respect to the multiple, complex, and interconnected forms of disadvantage that currently impede equity in maternal health utilization. Identifying and developing a deeper understanding of the mechanisms by which different predictors (such as age at first marriage) might impact utilization practices represents an important step towards addressing this challenge.

## Paper context

This paper explores the factors that impede or facilitate the utilization of healthcare services. Three specific utilization practices are explored in India’s most populated State Uttar Pradesh: registration of the pregnancy, receiving any antenatal care and if the delivery was at home. Predictors of utilization include literacy of the mother, husband's schooling, age at first marriage, and socio-economic factors. Knowledge of such predictors can help implement complex interventions that can incorporate equity considerations in healthcare utilization.
